# Supplementary figures and images for: CRIMSON: An open-source software framework for cardiovascular integrated modelling and simulation
Source: PLoS Comput Biol. 2021 May 10;17(5):e1008881. doi: 10.1371/journal.pcbi.1008881 (PMC8148362; doi:10.1371/journal.pcbi.1008881)

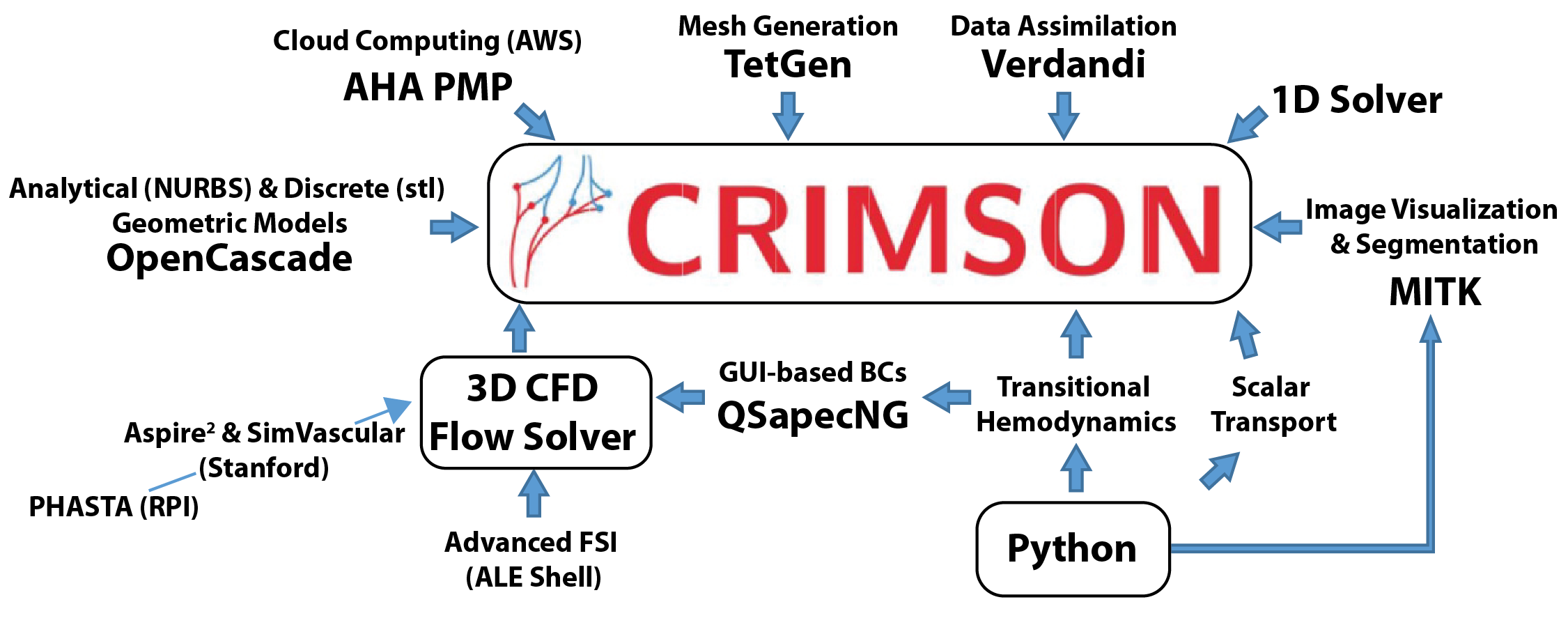

Supplement: S1 Fig — (PNG) [file pcbi.1008881.s001.png]
